# Serotyping of *Toxoplasma gondii* in Cats (*Felis domesticus*) Reveals Predominance of Type II Infections in Germany 

**DOI:** 10.1371/journal.pone.0080213

**Published:** 2013-11-07

**Authors:** Pavlo Maksimov, Johannes Zerweck, Jitender P. Dubey, Nikola Pantchev, Caroline F. Frey, Aline Maksimov, Ulf Reimer, Mike Schutkowski, Morteza Hosseininejad, Mario Ziller, Franz J. Conraths, Gereon Schares

**Affiliations:** 1 Institute of Epidemiology, Friedrich-Loeffler-Institut, Federal Research Institute for Animal Health, Greifswald - Insel Riems, Germany; 2 JPT, Peptide Technologies GmbH, Berlin, Germany; 3 Animal Parasitic Diseases Laboratory, USDA, ARS, ANRI, BARC-East, Beltsville, Maryland, United States of America; 4 Vet Med Labor GmbH, Division of IDEXX Laboratories, Ludwigsburg, Germany; 5 Institute of Parasitology, Vetsuisse Faculty, University of Bern, Bern, Switzerland; 6 Institute for Biochemistry & Biotechnology, Department of Enzymology, Martin-Luther-University Halle-Wittenberg, Halle (Saale), Germany; 7 Faculty of Veterinary Medicine, University of Shahrekord, Shahrekord, Iran; 8 Workgroup Biomathematics, Friedrich-Loeffler-Institut, Federal Research Institute for Animal Health, Greifswald - Insel Riems, Germany; University of California, Riverside, United States of America

## Abstract

**Background:**

Cats are definitive hosts of *Toxoplasma gondii* and play an essential role in the epidemiology of this parasite. The study aims at clarifying whether cats are able to develop specific antibodies against different clonal types of *T. gondii* and to determine by serotyping the *T. gondii* clonal types prevailing in cats as intermediate hosts in Germany.

**Methodology:**

To establish a peptide-microarray serotyping test, we identified 24 suitable peptides using serological *T. gondii* positive (n=21) and negative cat sera (n=52). To determine the clonal type-specific antibody response of cats in Germany, 86 field sera from *T. gondii* seropositive naturally infected cats were tested. In addition, we analyzed the antibody response in cats experimentally infected with non-canonical *T. gondii* types (n=7).

**Findings:**

Positive cat reference sera reacted predominantly with peptides harbouring amino acid sequences specific for the clonal *T. gondii* type the cats were infected with. When the array was applied to field sera from Germany, 98.8% (85/86) of naturally-infected cats recognized similar peptide patterns as *T. gondii* type II reference sera and showed the strongest reaction intensities with clonal type II-specific peptides. In addition, naturally infected cats recognized type II-specific peptides significantly more frequently than peptides of other type-specificities. Cats infected with non-canonical types showed the strongest reactivity with peptides presenting amino-acid sequences specific for both, type I and type III.

**Conclusions:**

Cats are able to mount a clonal type-specific antibody response against *T. gondii*. Serotyping revealed for most seropositive field sera patterns resembling those observed after clonal type II-*T. gondii* infection. This finding is in accord with our previous results on the occurrence of *T. gondii* clonal types in oocysts shed by cats in Germany.

## Introduction

*Toxoplasma gondii* is a zoonotic obligate intracellular parasite which causes toxoplasmosis in humans and animals. Felids are definitive hosts of this parasite and almost all warm-blooded mammals including humans and cats [1,2] can serve as intermediate hosts*.*

The population of *T. gondii* in Europe and North America is dominated by three clonal types (I, II and III), whereas the majority of characterized isolates from South America and Africa are genetically different from these canonical types. Most of the genotypes observed in Brazil are regarded as non-canonical or “atypical”. PCR-RFLP revealed mainly combinations of type I and III specific alleles [3]. This, however, does not mean that they represent sexual recombinants derived from canonical types but should rather be regarded as evolutionary separate lineages [4-6]. 

The *T. gondii* clonal type is regarded as a key-factor responsible for the clinical appearance of toxoplasmosis in outbred mice [7]. There is growing evidence that this may also apply to other intermediate hosts including humans [6,8,9]. Canonical and non-canonical *T. gondii* were associated with certain clinical appearances in humans [10-12]. However, the geographical distribution and dominance of particular *T. gondii* types as well as host genetic and immunity related factors may have biased prior studies [13-15]. For ocular toxoplasmosis, for example, it was demonstrated that most cases in South America were caused by non-canonical *T. gondii* [16], whereas a predominance of *T. gondii* type II was found in France [17,18]. However, Gilbert et al. (2008) [16] demonstrated that congenitally infected Brazilian children were five times more likely to develop ocular toxoplasmosis with more severe symptoms than congenitally infected children from Europe. McLeod et al. (2012) observed both *T. gondii* serotypes, II and NE-II (i.e., not exclusively serotype II), in cases of congenital toxoplasmosis in North America. However, the *T. gondii* serotype NE-II was more frequently found in certain demographic groups and was statistically associated with more severe cases of congenital toxoplasmosis [9]. These findings may suggest that the severity of human toxoplasmosis could be influenced by the genotype of *T. gondii* that has caused the infection. It is therefore epidemiologically relevant to determine the *T. gondii* types dominating in particular geographical areas and to compare the *T. gondii* types prevailing in clinical cases of toxoplasmosis in humans and animals [19]. 

The majority of typing studies on *T. gondii* in cats were performed by using DNA-dependent techniques [20-24]. However, most *T. gondii* DNA samples were obtained either from tissues/tissue cysts from euthanized cats or from oocysts isolated from feline fecal samples. It is difficult to obtain sufficient amounts of parasite DNA from host tissues and fluids even in cases of clinical toxoplasmosis. *T. gondii* DNA from subclinical cases – which would be of utmost importance for epidemiological studies on potential type-related effects – are not available. Serotyping allows not only the inclusion of clinical, but also of subclinical cases. This explains why typing *T. gondii* infections via the antibody response is attractive and has prompted a number of studies in the past. 

*T. gondii* infection causes a strong and often persistent humoral immune response with detectable antibody titers, independent of the clinical manifestations in the infected host [25,26]. 

Some of the *T. gondii* antigenic proteins are presenting sequence differences in the polypeptides expressed by different clonal types [10,27,28]. Kong et al. (2003) [10] demonstrated that the humoral response against *T. gondii* is partially type-specific, when the sites of clonal type-specific polymorphisms are used as peptide antigens. Based on these results several studies on the serotyping of *T. gondii* in humans using polymorphic synthetic peptides have been performed. The results suggested that it is possible to distinguish between type II- and non-type II-infection [10,27,29-32]. Xiao et al. (2009) identified peptides that could be also used to distinguish between type III- and type I-infections [31].

Cats play an important role in the epidemiology of *T. gondii* infection because they are definitive hosts of the parasite. They can excrete large numbers of environmentally resistant oocysts, which represent after sporulation one of the main infection sources for intermediate hosts [33]. Most of the *T. gondii* from cats were genetically characterized by PCR-RFLP and sequencing [34-37] after isolation via mouse bioassay using infected cat tissues or oocysts from faecal samples [20,21]. Since *T. gondii* infection in cats is normally asymptomatic, it is difficult to isolate the parasite or to detect oocyst shedding in healthy cats. However, infected cats usually develop antibodies against *T. gondii* within approximately 2 weeks after infection [33,38,39]. Consequently, serotyping could be an alternative method to estimate the prevalence of *T. gondii* types in cats. It was unknown whether serotyping of *T. gondii* in cats is possible. We therefore first investigated if cats were able to mount a specific serological response against canonical clonal types of *T. gondii*. A synthetic peptide-microarray was established and used to determine the *T. gondii* serotype of naturally infected cats in Germany. Polymorphic peptides identified for serotyping in cats could also be suitable for serotyping in other species including humans. 

## Materials and Methods

### Ethics Statement

All experiments in cats and mice carried out in USA had been approved by Beltsville Area Animal Care and Use Committee (BAACUC).

Experimental infection of cats carried out in Iran was followed as described by the Iranian animal rights organization [40] and was approved with respect to animal rights by the Ethic Committee of the Deputy for Research and High Education Affairs of the Veterinary Faculty of Shahrekord University (No. 122.5938-9). Details on the experimental infections conducted in Iran and the serological results of the kittens were published elsewhere [41]. Sera from cats in Germany and Switzerland were collected for routine veterinary diagnostic purposes and not for the purpose of research; consequently no ethical approval was needed.

### Selection of peptide sequences

A total of 101 *T. gondii* polymorphic peptide sequences, i.e. peptide sequences, which differed for at least two of the three canonical types I, II and III, were chosen to detect clonal type-specific antibodies in sera from *T. gondii*-infected animals. These included 54 peptide sequences from 15 *T. gondii* immunogens previously reported as clonal type-specific [10]. 

In addition, 47 of the 101 polymorphic peptides were selected based on *T. gondii* amino acid sequences partially published in Genbank. They included peptides from the dense granule proteins GRA6 (AAF60334; AAF60336; AAF60337) [27,42], GRA5 (sequences were taken from a publication, [27]), and GRA7 (ABE69193; EU157141; DQ459455) [43,44] as well as the surface antigen SAG2A (AAK50636; AAK50637; AAK50638; AAF79155) [45,46]. For the identification of polymorphic, i.e. type-specific amino acid (aa) sequences, protein sequence analysis and alignment was performed using the “MegAlign” tool provided by DNAStar software (DNASTAR, Inc; Madison; Wisconsin; USA). Polymorphic, 15 aa long peptide sequences containing B-cell epitopes were selected using the “Protean” tool of DNAStar (DNAStar Inc; Madison; Wisconsin; USA). Propensity scale methods and cut-offs implemented in this program were used to identify aa regions with potential B cell epitopes according to following criteria: (i) predicted alpha-helical structure (as determined by “Garnier-Robson plots” [47]), (ii) presence of proline residues, (iii) substantial content of hydrophilic amino acids (as determined by “Kyte-Doolittle hydropathy plots” [48]), (iv) high “antigenic index” using “Jameson and Wolf plots” [49] integrating flexibility parameters with hydropathy/solvent accessibility values, and (v) a high surface probability (“Emini’s surface probability plots” [50]), based on side-chain solvent accessibility values of the individual aa. Information on all peptide sequences selected for this study is presented in Table **S1**.

### Cat sera

#### Sera from cats infected with canonical *T. gondii*

In all, 17 cat sera specific for clonal type I, 3 sera specific for clonal type II and one serum specific for clonal type III were available as positive reference standards for the validation of peptides for *T. gondii* serotyping in cats. Sera obtained from cats inoculated with tissue cysts of mouse virulent non-canonical *T. gondii* isolates (n=7) were also included.

The serological status of the reference sera was determined in an immunofluorescence antibody test (IFAT) and by immunoblotting using *T. gondii* surface antigen 1 (TgSAG1) as antigen (as described below).

Except one type II serum, which was obtained from an immunocompetent 10-year-old male cat that had died of systemic toxoplasmosis and from which a *T. gondii* strain presenting a clonal type II specific allele pattern in PCR-RFLP was isolated (in this study referred to as TgCatSw1) [51] (Table **S2**), all positive reference standards derived from experimental infections as described in the following.

Sera from cats infected *per os* with tissue cysts at the Animal Parasitic Diseases Laboratory, Beltsville Agricultural Research Centre, Maryland, USA: All cats fed with tissue cysts were bled prior to infection and had no antibodies in a 1:25 serum dilution tested by the MAT [52]. The number of tissue cysts in the inocula was unknown as the cats were fed whole infected tissues. The cats were obtained from a *T. gondii*-free cat colony as described previously [37]. They were 3-5 months old at the time of the experiment [39]. 

Three cats were infected with *T. gondii* type I tissue cysts from the CT1 strain, two with the GT1 strain and one cat with the RH strain. Sera from these cats were collected on days 22, 35, 25, 29, 34 and 43 post infection. Two cats were experimentally inoculated with *T. gondii* type II tissue cysts of the TgSdCo1 *T. gondii* isolate and ME49 strain [53,54]. Sera from these animals were collected on days 49 and 29 post inoculation respectively. To validate clonal type III-specific peptides, a single serum was available from a cat experimentally infected with tissue cysts from mice infected with the VEG strain [55]. The serum was collected on day 23 post infection (Table **S2**). 

Sera from cats experimentally infected with tachyzoites at the Veterinary Faculty of Shahrekord University, Iran: Six 45±5-day-old clinically healthy kittens of both genders were infected intraperitoneally with 10^4^ *T. gondii* RH tachyzoites in sterile PBS as described previously [41]. In five of six kittens infected with RH tachyzoites, serum samples were collected on days 18 and 26 post infections. From one kitten, only a serum sample collected on day 26 post infection was available (Table **S2**). 

#### Sera from *cats* infected with non-canonical *T. gondii*

Three isolates were from Paraná, Brazil (TgCatBr1, 2, 5) [56] (Table **S2**). Three cats were experimentally infected each with one of these isolates at the Animal Parasitic Diseases Laboratory, Beltsville, Agricultural Research Centre, Maryland, USA. Sera were collected for further analysis on days 42 and 30 post infection (Table **S2**). Further sera were from three cats infected with *T. gondii* isolated from a wild black bear from Alaska (TgBbUS1) [57] and one serum derived from a cat experimentally infected with *T. gondii* isolated from a goat in the USA (TgGoatUS6) [58]. Serum samples within this group were collected on days 37, 24, 33, 43 and 36 post infection (Table **S2**).

#### Sera from naturally *T. gondii* seropositive cats

All field sera from cats used in this study (n=138) were collected during serological routine testing for *T. gondii* at Vet Med Labor GmbH, Division of IDEXX Laboratories, Ludwigsburg, Germany. Fifty two serum samples were negative for *T. gondii* antibodies in both serological tests (IFAT and TgSAG1-immunoblot), and were used for the validation of peptides as part of the negative reference standard in a ROC analysis. Eighty six samples were seropositive in both serological tests and further used to determine the clonal types naturally *T. gondii*-infected cats from Germany were carrying.

### IFAT

The *T. gondii* strain RH (Sabin, 1941) was cultivated and used for preparation of IFAT slides as described previously [58]. The test was performed as described for *N. caninum* [59] but with the following modification: Anti-cat IgG [H+L] produced in goat and coupled to FITC (102-095-003, ImmunoResearch Laboratories, West Grove, Pennsylvania, USA) diluted 1:50 in PBS, 0.2% Evans Blue was used to detect primary antibodies. A reciprocal titer of 200 was used as the positive cut-off titer.

### TgSAG1 immunoblot

Native TgSAG1 was affinity-purified as described [58]. The identity of the purified protein was confirmed using monoclonal antibodies against TgSAG1 (IgG2a P30/3 [ISL, Paignton, UK]). Detection of antibodies against TgSAG1 was performed essentially as described [60] with a few modifications. Briefly, cat sera were diluted 1:100 and the conjugate (Peroxidase-conjugated AffiniPure Goat anti-cat IgG [H+L], 102-035-003, Jackson ImmunoResearch, West Grove, Pennsylvania, USA) was diluted 1:500. Reactivity with a 30 kDa band was regarded as a *T. gondii*-positive reaction Two sera obtained from an IFAT-positive and an IFAT-negative cat were used as controls. 

### Peptide-microarray

Peptides were synthesized and printed on peptide-microarray slides (i.e. modified glass-slides) by JPT Peptide Technologies GmbH, Berlin, Germany, essentially as described [30].

Cat serum samples were processed on peptide-microarrays as described [61] with a few modifications. Serum samples (60 µl/well), diluted 1:100 in blocking solution (PBS, 0.05% Tween 20, 0.2% I-Block [Applied Biosystems, Bedford, MA, USA]), were incubated at 37°C for 1 h and washed seven times for 3 min with PBS-T (PBS, pH 7.2; 0.5% Tween 20) at room temperature. Conjugate (Biotin-SP-conjugated AffiniPure goat anti-cat IgG, Fc Fragment Specific, 102-065-008, Jackson ImmunoResearch Laboratories, West Grove, Pennsylvania, USA) diluted in blocking solution 1:500 (1 µg/ml) was added to the wells (60 µl/well), incubated at 37°C for 30 min, and washed as indicated above. Cy^Tm^5-conjugated streptavidin (016-170-084, Jackson ImmunoResearch Laboratories, West Grove, Pennsylvania, USA) diluted in blocking solution 1:500 (1 µg/ml) was added to the wells (60 µl/Well), incubated at 37 °C for 30 min and washed as described, followed by three additional washing steps, 1 min each, with sterile-filtered MilliQ water. Afterwards, the microarrays were spun dry for 10 s using a slide spinner (DW-41MA-230, Qualitron Inc/Eppendorf, Berzdorf, Germany).

Scanning and evaluation of microarrays, as well as data extraction was performed as described [30].

### Microarray data analysis

To analyze the raw data (median of signal intensity) in GPR (GenePix Results) files, index values (IVs), as well as the means of the IVs for each peptide triplicate per block (mean sample index value, MSIV) were recovered as described previously using R (R version 2.14.1 (2011-07-08) Copyright (C) 2011; ISBN 3-900051-08-9; http://CRAN.R-project.org/) [30,61-63]. The peptide-microarrays used in this study failed to meet the criteria required for submission under MIAME based public databases, because the data type of biomolecular interaction and parameters studied, the protocols as well as the type of information extracted from the microarray experiment differed from standard DNA microarray experiments [30,61,64,65]. Therefore MSIVs for all sera and peptides are presented as supplemental material (Table **S3**).

### Statistical analysis

For statistical analysis and graphical presentation of results, the R program environment was used (R version 2.14.1 (2011-07-08) Copyright (C) 2011; ISBN 3-900051-08-9; http://CRAN.R-project.org/ ). 

To select peptides appropriate for serotyping and to establish individual cut-offs for each peptide, a Receiver Operating Characteristics (ROC) analysis was conducted using the R-package ‘‘DiagnosisMed’’. 

For the ROC based selection of peptides derived from polymorphic clonal type-specific regions, reactions of reference sera derived from cats infected with a homologous type of *T. gondii* were used as a “positive reference standard”. In contrast, *T. gondii* negative sera and sera from cats infected with a heterologous clonal type of *T. gondii* were used as a “negative reference standard”. ROC analysis was performed using these reference standards and based on literature information [32,66] peptides with an Area-Under-ROC-Curve (AUC) value of ≥ 0.7 were considered to have enough discriminatory power for serotyping [67]; the higher the AUC the better a peptide discriminates between reference positive and negative sera. ROC analysis was also used to define individual peptide-specific cut-offs, at which the sum of diagnostic sensitivity and specificity for individual peptides reached its optimum, i.e. was as high as possible. These cut-offs for individual peptides were used to define positive and negative peptide reactions, to determine typing sensitivity and specificity and to determine the proportion of false positive typing reactions among the *T. gondii*-positive cat reference sera infected with a heterologous *T. gondii* type in relation to peptide specificity and serologically negative cat sera. Peptides with a proportion of false positive reactions among these sera of > 45% were excluded from further analysis. Positive and negative reactions of the finally selected peptides were used for further frequency and Post-Hoc-Test (LSD [Least-Significant-Difference]) of ANOVA analyses. 

Frequency analysis (Log-linear model, Chi-Square) for serotyping data was performed with the R-package “vcd”, which was also used to visualize the results in mosaic plots as described previously [28,30,68]. 

Serum-peptide reactions (mean sample index values, MSIVs) were cut-off normalized (CN) by subtracting the peptide-specific cut-off values from MSIV resulting in a value called CN-MSIV. This was done to achieve a better visualization of positive and negative serum-peptide reactions, e.g. positive reactions led to values ≥ 0 and negative results to values < 0. To perform multiple comparisons of CN-MSIV means between peptide groups or between single peptides, a Post-Hoc-Test (LSD [Least-Significant-Difference]) on ANOVA results was applied using the R package “agricolae” [30,61]. 

To analyse whether peptide patterns recognized by individual sera cluster in different groups, we performed explorative data analysis applying the artificial neural network-based Selforganizing Kohonen Network/Selforganizing Kohonen Maps (SOM) method [69-72]. In the present study we used supervised XY-fused Selforganizing Kohonen Network analysis to find a relationship between the input data (measurements of serum-peptide reactions and peptide groups) presented by X-map and output data (cat sera groups) presented by Y-map. XY-fused supervised Kohonen network (XYF-SKN) analysis was applied using the R package “kohonen” [69,70]. We used a SOM grid of 4×2 units (nodes). The topology of the grid was hexagonal. The complete data set was presented 2000 times to the network. For this analysis, serum-peptide reactions were used with original MSIVs. 

## Results

### Confirmation of the *T. gondii* serological status of cat sera

IFAT was used to determine the serological status of cats. Within the group of sera regarded as *T. gondii* negative (n=52), the reciprocal IFAT titres ranged from <25 to 100. Among the sera regarded as positive (n=114) reciprocal IFAT titres ranged from 200 to 51200 (Table **1**, Table **S3**). IFAT results were confirmed by the TgSAG1 immunoblot. In 114 of 114 (100%) IFAT positive field and reference sera antibodies to TgSAG1 were detected, while 52 of 52 (100%) of the IFAT negative field sera were negative in the TgSAG1 immunoblot. 

**Table 1:** Positive and negative TgSAG1 immunoblot reactions versus reciprocal *Toxoplasma gondii* IFAT titers in feline field and reference sera (n=166).

	Reciprocal *Toxoplasma gondii* IFAT titre*												
TgSAG1 immunoblot	<25	25	50	100	200	400	800	1600	3200	6400	12800	25600	51200
Negative	12	25	8	7	0	0	0	0	0	0	0	0	0
Positive	0	0	0	0	2	2	5	17	26	15	19	22	6

* Positive reciprocal IFAT cut-off > 200

### Prediction of 47 novel potential polymorphic epitopes

In addition to 54 polymorphic peptides previously described [10], 47 novel polymorphic, i.e. type-specific, peptides derived from GRA5, GRA6, GRA7, SAG2A protein sequences were selected using a propensity scale method to extend the peptide panel for the serotyping of *T. gondii* clonal types by peptide-microarray using cat sera.

Based on the GRA5 protein sequences available for the clonal lineages I, II, and III 9 potentially type-specific peptide sequences were identified (Table **S1**). Compared to the type I GRA5 (RH strain), six aa substitutions were observed in type II GRA5 (K76 strain) and three aa changes in type III GRA5 (VEG strain) [27]. All except one aa substitution in GRA5 were located in the N-terminal hydrophilic region of GRA5. The selected GRA5 peptide sequences did not meet all the criteria mentioned in the Materials and Methods section for the selection of peptides (Table **S1**): Proline residues were missing in all GRA5 peptide sequences, and only three of the selected GRA5-derived peptides we located in putative α-helical regions (according to the “Garnier-Robson plot”). The “Jameson-Wolf antigenicity index” suggested, however, that the chosen peptides were located in antigenic regions and “Emini’s surface probability plot” showed a high surface-probability for all the selected peptide-sequences. All sequences were located in hydrophilic regions of the GRA5 protein according the “Kyte-Doolittle hydropathy plots” (Table **S1**).

In the GRA6 aa sequences, 21 polymorphic positions were identified. In comparison with type I GRA6 (RH strain), the protein sequence revealed eight aa substitutions and six aa insertions in type II (ME49 strain), while seven aa substitutions were detected in type III GRA6 (NED strain). All peptides met most selection criteria (Table **S1**). Only three peptides were located in a region with α-helical properties. Five peptide sequences contained no proline residues. The selected peptides were located at both, C- and N- terminal regions (Table **S1**). 

For GRA7, 13 polymorphic peptide sequences were selected. All peptide sequences were located near the C-terminus. In comparison to GRA7 type I (RH strain), eight aa substitutions were observed in type II (BEVERLEY strain) and 15 aa substitutions in type III (NED strain) (Table **S1**). All GRA7 peptides were derived from antigenic, hydrophilic regions. They were selected from regions putatively located on the protein surface or in putative α-helical regions. Four peptides lacked proline residues. 

For the SAG2A protein, four polymorphic peptides from the C-terminal region were chosen. The selected peptide regions met the criteria of antigenicity, hydrophilicity, surface probability and presence of proline residues. However, the peptides failed to present an α-helical structure (Table **S1**). Peptides were derived either from a polymorphic protein region common for clonal types I and III (strains S48, NED) or from a region specific for clonal type II (strains LGE96-1, BEVERLEY).

### Selection of 24 peptides appropriate for *T. gondii* serotyping in cats

In total, 101 peptides presenting single type-specific polymorphisms (I [n=27], II [n=29] and III [n= 21]) as well as common polymorphisms for two of three clonal types simultaneously (I/II [n=6], I/III [n=12], II/III [n=6]), were used initially. For the selection of peptides suitable for *T. gondii* serotyping individual peptides were first subjected to a ROC analysis. Those peptides for which ROC analysis revealed a diagnostic capacity were further analyzed by ANOVA and LSD-Post-Hoc-Test. 

For each group of peptides specific for a particular clonal type, an individual set of reference sera was used in ROC analysis, which included sera from *T. gondii* serologically negative cats (n=52) and sera from experimentally-infected cats (n=21). In the individual ROC analyses, 40 of the 101 analyzed peptides yielded on AUC value ≥ 0.7 (Table **S4**) and were thus included in a second phase of selection. In this phase, peptides were excluded which showed a proportion of false positive reactions of > 45% either in negative field sera or in the sera of cats infected with a heterologous *T. gondii*-type in relation to peptide specificity (Table **S4**). Further three peptides were excluded during ANOVA and LSD-Post-Hoc-Test analysis because they were either recognized nonspecifically (i.e. showed significantly higher reaction intensities in heterologous or negative sera than in sera with homologous specificity) or there were no statistically-significant differences in reactivity between the different groups of cat sera according to the LSD-Post-Hoc-Test. 

A total of 24 remaining peptides were recognized by sera with homologous specificity (Table **S4**). Diagnostic specificity using the “negative reference standard”, i.e. the level of restriction of reactions to sera from animals infected with a clonal type homologous to the type-specificity of the aa sequence of this individual peptide, ranked from 60% to 98%. The proportion of false positive reactions among the sera from animals infected with a clonal type heterologous to the type-specificity of the peptide ranged from 0% to 44.4% (Table **S4**). The proportion of false positive reactions in serological negative filed sera ranged from 0% to 40.4% (Table **S4**). Diagnostic sensitivity using positive sera from cats infected with the homologous type of *T. gondii* in relation to peptide specificity ranged between 5.9% and 100% (Table **S4**). The peptides selected for serological typing were derived from GRA1, 3, 5, 6, 7 and SAG2A proteins. Nine of the 24 peptides were novel, while 15 had previously been published [10]. In the peptide panel established in the present study, the type-specificities were almost equally distributed over the three clonal types of *T. gondii* (type I: 9 peptides, type II: 9 peptides, type III: 11 peptides). The aa sequences of the 24 peptides showed the following specificities in detail: type I (n=4), type II (n=8), type III (n=7), type I/II (n=1) and type I/III (n=4). 

### Serotyping in cats infected with known *T. gondii* type

#### Clonal type I-infected cats

**Figure 1:**
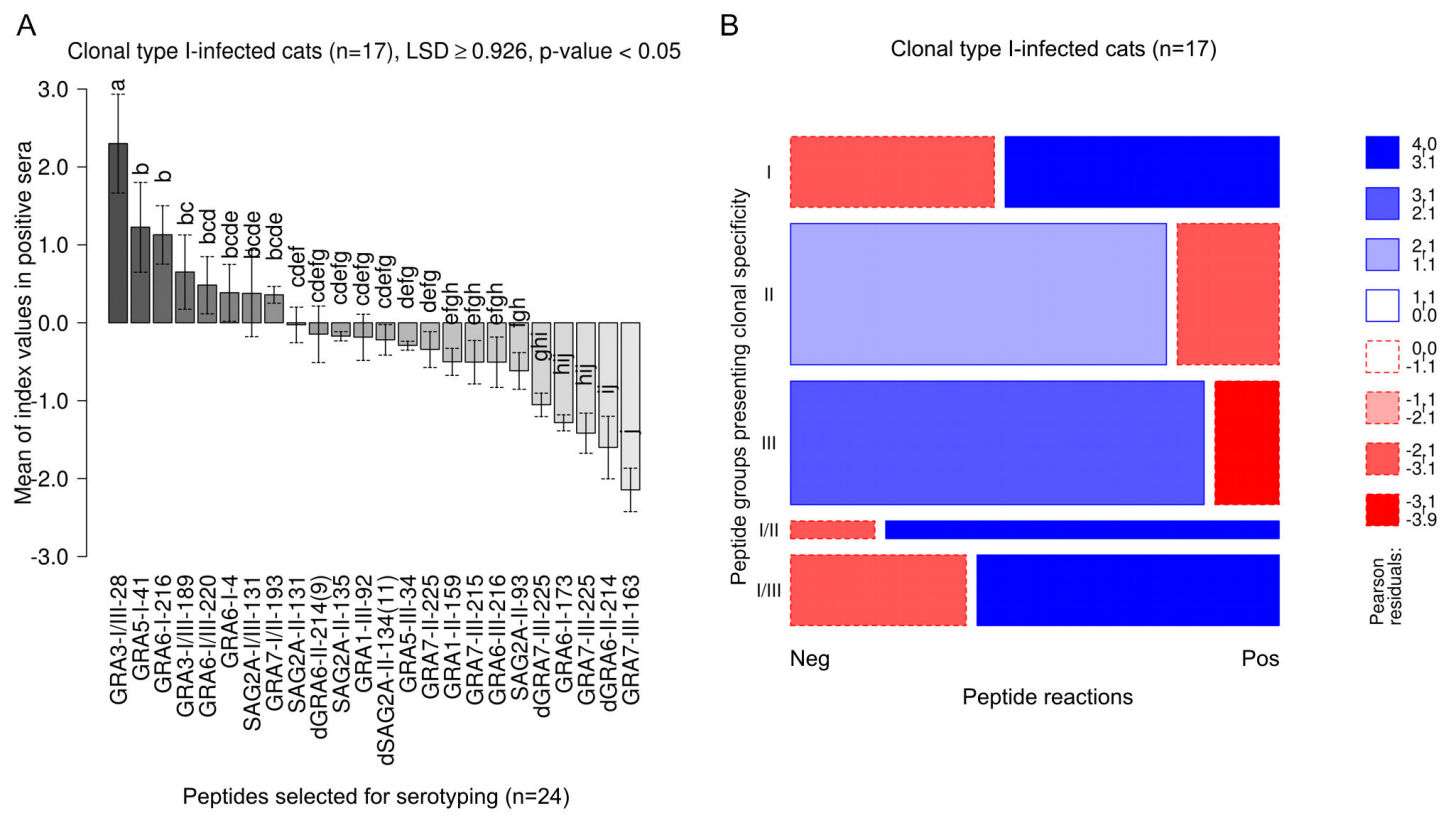
In *Toxoplasma gondii* type I-infected cats reactions against type I, I/II and I/III specific peptides are strongest and are overrepresented in number. Intensities (MSIVs) by which clonal type I-infected cats reacted with individual peptides were analyzed using ANOVA and the Least Significant Difference (LSD)-Post-Hoc-Test (A). Whiskers represent 95% confidence intervals of the means of MSIV (bars). The differences between the means of MSIVs were regarded as statistically significant, when they were equal or higher than the LSD values. Different letters above the whiskers indicate significant differences between the mean intensities in the LSD-Post-Hoc-Test. To evaluate whether positive or negative serum reactions against clonal type-specific peptide cohorts were over- or underrepresented in cats infected with *T. gondii* clonal type I, a log-linear model analysis was used and the results presented in a mosaic plot (B). The size of each box in the mosaic plot corresponds to the observed frequencies of positive (Pos) and negative (Neg) peptide reactions as well as the number of analyzed peptides within each peptide cohort. Pearson residuals represent standardized deviations of observed from expected values. The Pearson residuals 0-2 with solid blue line indicate that the number of positive or negative reactions is higher, but not statistically significantly higher than expected (Pearson chi-squared p-value < 0.1). Blue scale shadings suggest the statistically significant rejection of the null hypothesis, i.e. overrepresentation of reactions against particular peptide groups (Pearson residuals (>2), Pearson chi-squared p-value < 0.05). Dashed red lines indicate an underrepresentation of positive or negative peptide reactions which is not statistically significant. Red scale shadings suggest a statistically significant rejection of the null hypothesis, i.e. underrepresentation of peptide reactions within the analyzed peptide group (Pearson residuals (<-2) Pearson chi-squared p-value, 0.05).

The mean CN-MSIVs by which peptides with clonal type I-specific aa sequences (I, I/II, I/III) were recognized by sera of clonal type I-infected cats (n=17) were significantly higher than the mean CN-MSIVs by which peptides with type II- or III-specific, i.e. heterologous aa sequences were recognized by the same sera (Table **2**, ANOVA, LSD ≥ 0.523, p-value < 0.05). The GRA3-I/III-28 peptide was recognized by the significantly highest MSIVs in this group (Figure **1** **[A**]), followed by GRA5-I-41, and GRA6-I-216 (ANOVA, LSD ≥ 0.926, p-value < 0.05). The lowest anti-clonal type I-specific reactivity was observed for peptide GRA6-I-173 (Figure **1** **[A**]). The diagnostic sensitivity of individual peptides presenting clonal type I-specificity (I, I/II, I/III) ranged from 5.9% to 94.1% and the diagnostic specificity from 64.2% to 98.2% (Table **S4**). 

**Table 2:** Means of cut-off normalized mean reaction values in peptide and cat groups analyzed by ANOVA/LSD Post-Hoc-Test.

Cat groups* (Least significant difference, LSD)	Means of CN-MSIV within peptide groups (95% Cl)**				
	I	II	III	I/II	I/III
I (*≥ 0.523*)	**0.37 (0.008…0.81**)	-0.45 (-0.64…-0.26)	-0.87 (-1.09 …0.65)	**0.36 (0.15…0.57**)	**0.95 (0.42…1.49**)
II (*≥ 1.034*)	-1,04(-1.31…-0.77)	**0.79 (0.15…1.43**)	-1.12 (-1.70…-0.54)	**0.92 (0.15…1.69**)	-1.15 (-1.62…-0.67)
III (*≥ 1.129*)	-0.59 (-1.37…-0.20)	-0.29 (-0.97…-0.39)	0 (0…0)	-0.27 (0…0)	**1.55 (0.31…2.79**)
N (*≥ 0.259*)	-0.73 (-0.84…-0.61)	**-0.11 (-0.24…0.03**)	-1.19 (-1.33…1.05)	**0.09 (0.0004…0.18**)	-1.08 (-1.27…-0.89)
A (*≥ 0.902*)	-0.23 (-1.01…0.54)	0.12 (-0.33…0.57)	-0.56 (-0.98…-0.14)	0.05 (0.17…0.28)	**2.37 (1.58…3.16**)

* Cat groups: I, cats infected with *Toxoplasma gondii* clonal type I (n=17); II, cats infected with *T. gondii* clonal type II (n=3); III, cat infected with *T. gondii* clonal type III (n=1); N, naturally *T. gondii*-infected cats (n=86); A, cats infected with non-canonical *T. gondii* types (n=7); ** Peptide groups: I, II, or III, peptides with *T. gondii* type I, II, or III specific amino acid (aa) sequences; I/II, peptides with aa sequences specific for both, type I and II; I/III, peptides with aa sequences specific for both, type I and III; CI: confidence interval; CN-MSIV: Cut-off normalized mean reaction values (reaction value – cut-off value = CN-MSIV); In bold: statistically significant higher mean of CN-MSIVs according to ANOVA/LSD Post-Hoc-Test

The prevalence of positive and negative serum-peptide reactions within the group of type I-infected cats was analysed using a log-linear model. The resulting contingency tables and deviations from the hypothesis of independence were visualized by mosaic plots (Figure **1** **[B**]). Positive clonal type I-specific peptide reactions were quantitatively overrepresented (Pearson residuals > 3, p-value < 0.01) among sera of cats infected with type I *T. gondii*. In addition, type I/II- and I/III-specific peptide reactions were also statistically significantly overrepresented as indicated by Pearson residuals > 3 (p-value < 0.01). All type II- and III-specific positive peptide reactions were underrepresented (Pearson residuals < -2, and < -3, p-value < 0.05 and 0.01 respectively) among sera from type I-infected cats (Figure **1** **[B**]).

#### Clonal type II infected cats

**Figure 2:**
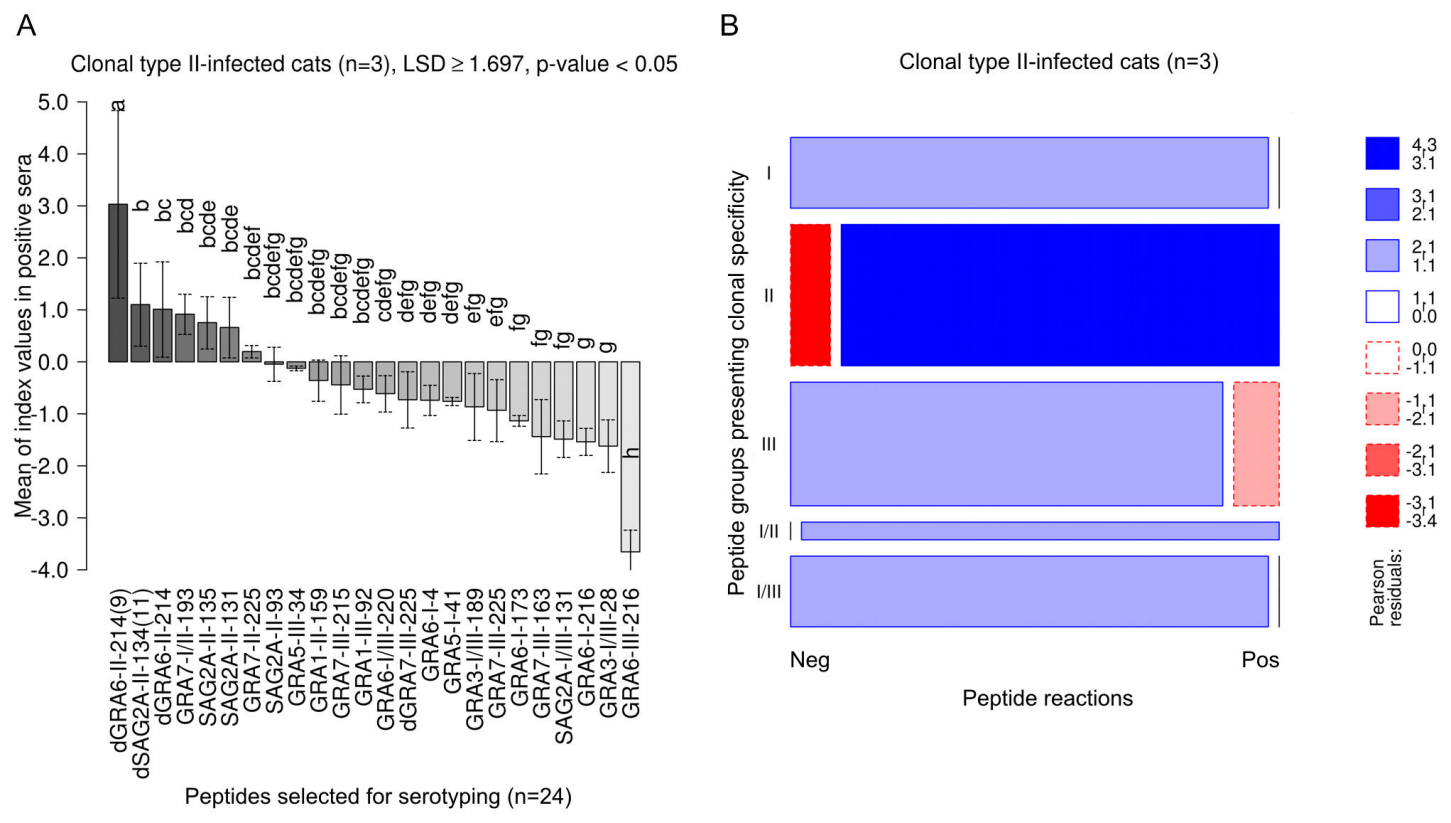
In *Toxoplasma gondii* type II-infected cats reactions against type II and I/II specific peptides are strongest and overrepresented in number. Intensities (MSIVs) by which clonal type II-infected cats reacted with individual peptides were analyzed using ANOVA and the Least Significant Difference (LSD)-Post-Hoc-Test (A). To evaluate whether positive or negative serum reactions against clonal type-specific peptide cohorts were over- or underrepresented in cats infected with *T. gondii* clonal type II, a log-linear model analysis was used and the results presented in a mosaic plot (B). Detailed explanations of [A] and [B] are provided in Figure 1.

Analysis of sera from cats infected with clonal type II *T. gondii* (n=3) revealed that peptides presenting type II-specific aa sequences (II, I/II) were recognized by significantly higher mean CN-MSIVs as compared to those peptides with heterologous specificity, i.e. clonal type I or III (Table **2**; ANOVA, LSD ≥ 1.034, p-value > 0.05). The dGRA6-II-214(9) peptide was recognized by the significantly highest mean of CN-MSIVs (Figure **2** **[A**]), as compared to the remaining peptides used for serotyping (ANOVA, LSD ≥ 1.697, p-value < 0.05). The lowest means of clonal type II-specific CN-MSIVs were observed in GRA7-II-225, SAG2A-II-93 and GRA1-II-159. Peptides specific for clonal type I or III were recognized by markedly lower intensity as compared to those peptides presenting clonal type II specificity (ANOVA, LSD ≥ 1.697, p-value < 0.05) (Figure **2** **[A**]). The diagnostic sensitivity of peptides with clonal type II specificity (II, I/II) ranged between 66.7% and 100% and their typing specificity ranged from 60% to 98.6% (Table **S4**).

Estimation of the prevalence of positive peptide reactions by log-linear modelling showed a statistically significant overrepresentation of clonal type II- (Pearson residuals > 3, p-value < 0.01) as well as clonal type I/II-specific peptide reactions (Pearson residuals > 1, p-value < 0.1). In contrast, reactions against peptides with clonal type I- or III-specific sequences were underrepresented (Pearson residuals < -1, p-value < 0.1) in cats infected with clonal type II *T. gondii* (Figure **2** **[B**]).

#### Clonal type III-infected cat

**Figure 3:**
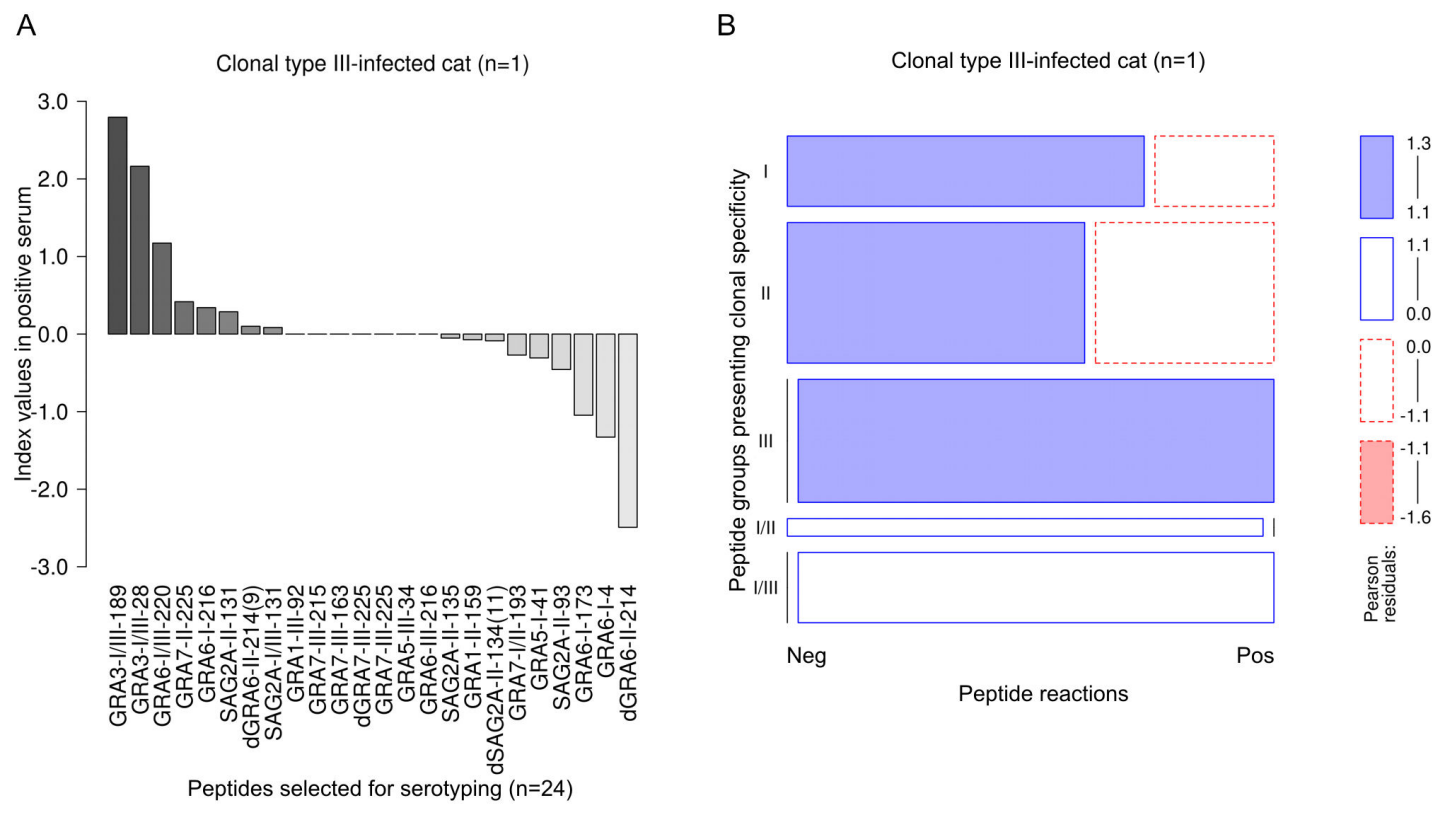
In a *Toxoplasma gondii* type III-infected cat type III and I/III specific peptides were recognized strongest and were overrepresented in number. Intensities (MSIVs) by which clonal type III-infected cats reacted with individual peptides were analyzed using ANOVA and the Least Significant Difference (LSD)-Post-Hoc-Test (A). To evaluate whether positive or negative serum reactions against clonal type-specific peptide cohorts were over- or underrepresented in a cat infected with *T. gondii* clonal type III, a log-linear model analysis was used and the results presented in a mosaic plot (B). Detailed explanations of [A] and [B] are already given in Figure 1.

The significantly highest CN-MSIVs of a serum from a cat infected with clonal type III *T. gondii* were observed in peptides with type I/III-specific aa sequences, followed by the CN-MSIVs observed in the group of clonal type III-specific peptides (Table **2**, ANOVA, LSD ≥ 1.128604, p-value < 0.05). The highest CN-MSIVs were observed in the peptides GRA3*-*I/III-189, GRA3-I/III-28 and GRA6-I/III-220. All peptides with clonal type III-specific sequences were recognized as positive, but reactions were close to the peptide-specific cut-offs (Figure **3** **[A**]). 

Four peptides with heterologous specificity (GRA7-II-225, GRA6-I-216 and SAG2A-II-131) were also recognized by the serum. However, the MSIVs recorded for these peptides were markedly lower (Figure **3** **[A**]) as compared to peptides with a homologous aa sequence specificity. 

Since only one reference serum specific for clonal III was available, it was not possible to establish a statistically significant log-linear model for the prevalence of positive or negative peptide reactions. Nevertheless, clonal type III- as well as I/III-specific positive peptide reactions were quantitatively overrepresented (Pearson residuals > 1, p-value < 0.1), whereas positive reactions from clonal type I-, II- and I/II-specific peptides were underrepresented (Figure **3** **[B**]). 

### Clonal type II-specific reactions dominate in naturally infected cats

Sera collected from naturally *T. gondii* seropositive cats from Germany (n=86) showed the strongest reactions (i.e. highest CN-MSIVs) with peptides of aa sequences homologous to clonal type II (II and I/II) as compared to reactions with peptides of clonal type I- or III-specific aa sequences (Table **2**; ANOVA, LSD ≥ 0.259, p-value < 0.05). 

**Figure 4:**
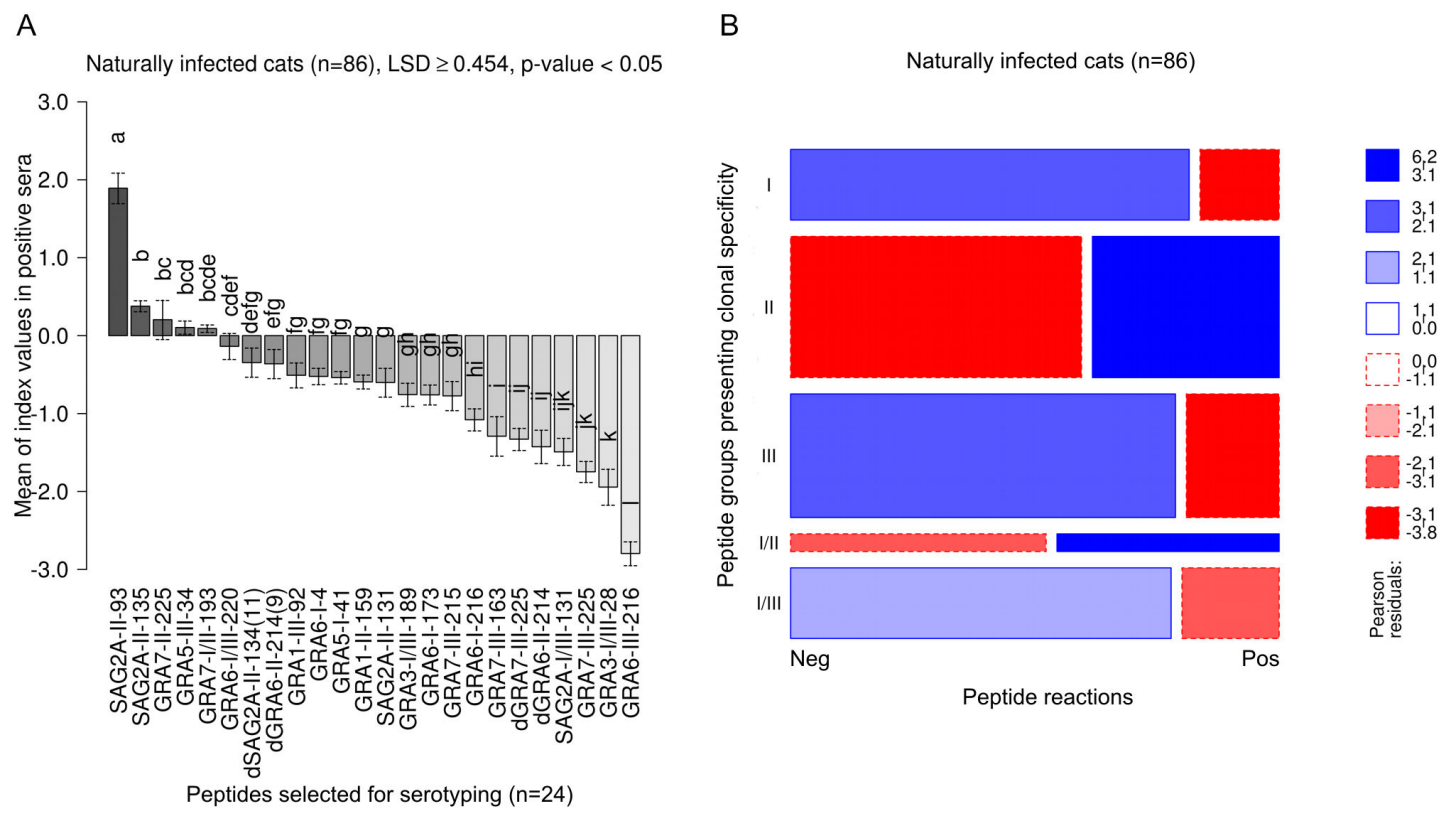
In naturally *Toxoplasma gondii* seropositive cats strongest reactions were observed against type II and I/II-specific peptides and reactions against these peptides were overrepresented. Intensities (MSIVs) by which naturally infected cats reacted with individual peptides were analyzed using ANOVA and the Least Significant Difference (LSD)-Post-Hoc-Test (A). To evaluate whether positive or negative serum reactions against clonal type-specific peptide cohorts were over- or underrepresented in naturally *T. gondii*-infected cats, a log-linear model analysis was carried out and results presented as mosaic plot (B). Detailed explanations of [A] and [B] are already given in Figure 1.

The highest CN-MSIVs were observed for peptide SAG2A-II-93 followed by SAG2A-II-135 and GRA7-II-225, which were both also recognized by significantly higher positive CN-MSVIs as compared to most remaining peptides, including GRA5-III-34 and GRA7-I/II-193 and GRA6-I/III-220 (ANOVA, LSD ≥ 0.454, p-value < 0.05) (Figure **4** **[A**]). 

SAG2A-II-93 was the peptide recognized by the highest number of *T. gondii* seropositive field sera (n=67, [77.9%]), followed by SAG2A-II-135 (n=60 [69.7%]), GRA7-II-225 (n=40, [46.5%]) and SAG2A-II-131(n=26, [30.2%]) (Table **S4**).

Log-linear model analysis revealed that reactions with peptides displaying clonal type II-specific aa sequences (II, I/II) were statistically significantly overrepresented (Pearson residuals > 3.1, Chi-squared p-value < 0.01) (Figure **4** **[B**]), whereas positive clonal type I- or III-specific peptide reactions were significantly underrepresented (Pearson residuals < 2.1, Chi-squared p-value < 0.05 (Figure **4** **[B**]).

### Cats infected with non-canonical *T. gondii* recognized mainly type I/III specific peptides

Cats infected with non-canonical *T. gondii* showed the strongest reactions, i.e. the highest mean CN-MSIVs, against peptides presenting aa sequences specific for type I and III (Table **2**; type I/III peptides). Statistically significant differences were not observed among the clonal type I-, II- ,III- and I/II-specific peptide groups (Table **2**; ANOVA, LSD ≥ 0.9, p-value < 0.05). 

**Figure 5:**
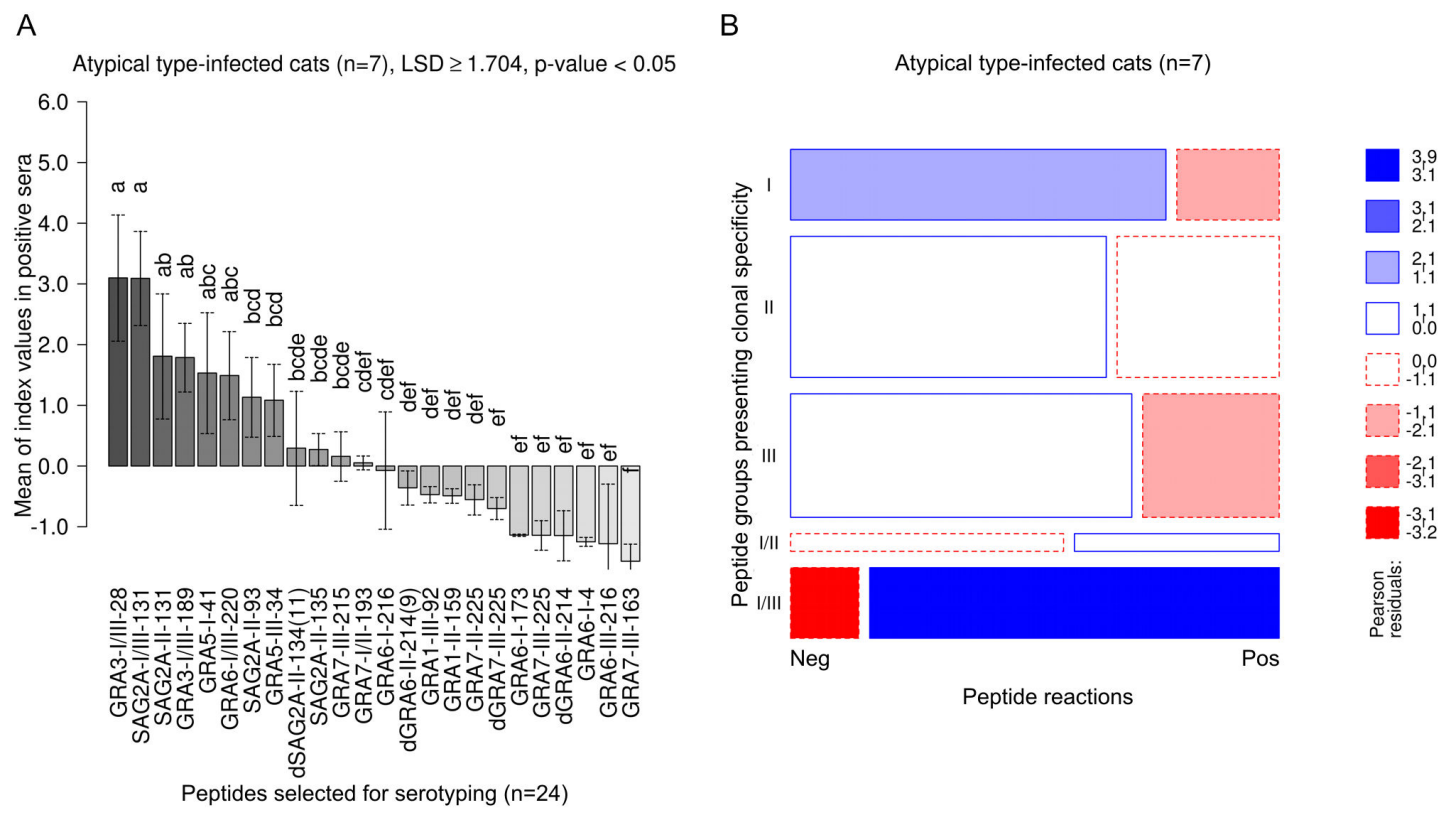
In cats infected with non-canonical *Toxoplasma gondii* strongest reactions were observed against type I/III specific peptides and the number of reactions against these peptides were overrepresented. Intensities (MSIVs) by which non-canonical type-infected cats reacted with individual peptides were analyzed using ANOVA and the Least Significant Difference (LSD)-Post-Hoc-Test (A). To evaluate whether positive or negative serum reactions against clonal type-specific peptide cohorts were over- or underrepresented in cats infected with atypical *T. gondii*, a log-linear model analysis was used and the results presented in a mosaic plot (B). Detailed explanations of [A] and [B] are already given in Figure 1.

Peptides GRA3-I/III-28 and SAG2A-I/III-131 were recognized with the highest mean CN-MSIVs. Lower CN-MSIVs were observed in SAG2A-II-131, GRA3-I/III-189, GRA5-I-41 and GRA6-I/III-220; however, the differences in reaction intensities were not statistically significant when compared with those against GRA3-I/III-28 and SAG2A-I/III-131. The remaining peptides were recognized in average by significantly lower index values (LSD ≥ 1.704, p-value < 0.05) (Figure **5** **[A**]). 

GRA3-I/III-189 peptide was recognized by all sera (100%), while 6 of 7 sera (86%) detected SAG2A-I/III-131and GRA3-I/III-28 (Table **S4**). 

Log-linear analysis of positive and negative peptide reactions among clonal type-specific peptides revealed that reactions with peptides containing sequences specific for both, type I and III, were statistically significantly overrepresented (Pearson residuals >3, p-value < 0.01), whereas clonal type I- and III-specific peptide reactions were underrepresented (Pearson residuals <-1, p-value < 0.1). No significant differences were observed in the frequency of positive and negative reactions among clonal type II- and I/II-specific peptide cohorts (Pearson residuals -1< 0 < 1, p-value > 0.1) (Figure **5** **[B**]).

### Naturally seropositive cats group together with type II-infected cats in a XY-fused supervised Kohonen network

Each tested serum recognized several peptides with a different MSIV. To detect similarities among the tested sera in the recognized peptide patterns, explorative analysis of the data using a XY-fused supervised Kohonen network (XYF-SKN) was performed. 

**Figure 6:**
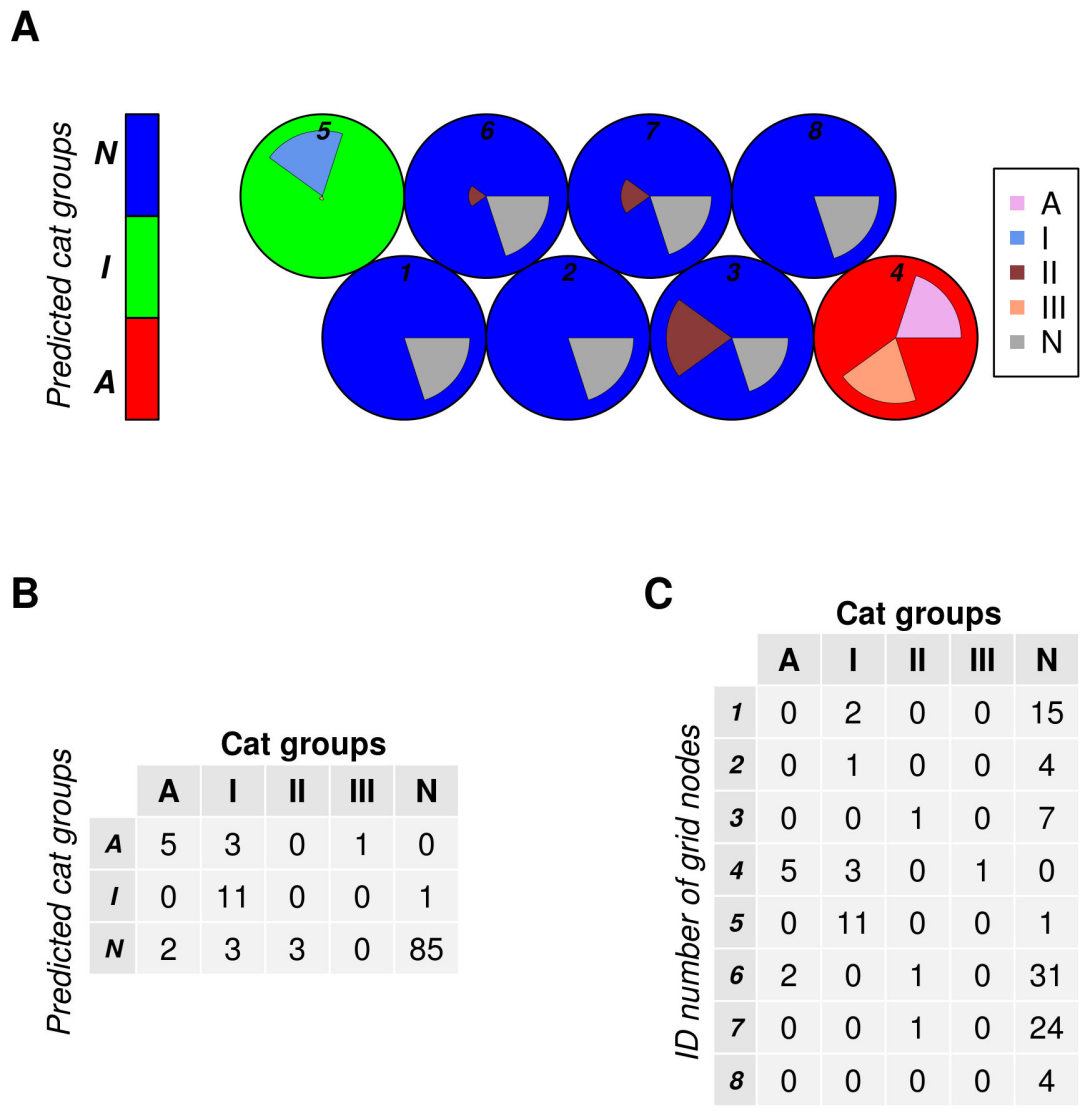
Naturally *Toxoplasma gondii* seropositive cats and cats with a known clonal type II infection recognized similar peptide patterns. To explore whether there are particular patterns of anti-peptide reactions among all serologically positive *T. gondii* cat sera, a XY-fused Selforganizing Kohonen Network analysis (XYF-SKN) was performed. Figure (A) presents the clustering of various groups of *T. gondii* positive cat sera in a Y-map. Most of the sera derived from cats infected with non-canonical *T. gondii* types (A), infected by clonal types I, II or III (I, II, or III), or naturally infected cats (N) clustered in the grid nodes *1* to *8* either together (type III- and atypical type-infected [node *4*] as well as the type II and naturally infected cats [nodes *1* to *3* and *6* to *8*]) or separately (cats infected with type I *T. gondii* [node *5*]). Three cat groups were predicted using XYF-SKN, e.g. a cat group infected with non-canonical and type III *T. gondii* strains (*A*), a clonal type I infected group (*I*) and a naturally-infected cat group (*N*). Predicted cat groups are presented by different background colours of grid nodes, e. g ***A*** by red, ***I*** by green and ***N*** by blue. Figure (B) shows the number of group-specific sera within predicted clusters (*A*, *I* and *N*), further confirming that sera of naturally infected cats clustered mainly with type II reference sera. Figure (C) shows the number of group-specific sera within eight grid nodes in Figure (A).

The Y-map of SKN is presented in the Figure **6** **[A**], where sera from five cat groups (i.e. three groups consisting of reference sera for type I, II or III, one group of sera from non-canonical type-infected cats and one group of sera from cats naturally infected with *T. gondii*) are clustered according to their similarity in peptide reactivity.

XYF-SKN classification analysis of serum-peptide reactions predicted three cat groups consisting mainly of animals infected with non-canonical and clonal type III *T. gondii* (***A***, red), clonal type I (***I***, green) or with natural infection (***N***, blue) (Figure **6** **[A**]**, [B**]). Group ***N*** (Figure **6** **[A**]**, [B**], blue) consisted of six nodes (1-3 and 6-8) with 85 (98.8%) sera of naturally infected cats and of all three sera from cats infected with *T. gondii* type II. Three sera from cats infected with *T. gondii* clonal type I and two sera collected from cats infected by non-canonical *T. gondii* fell also into this group. The second group ***I*** (Figure **6** **[A**]**, [B**], green) consisted of a single grid node (5) with 11 type-I-sera and one serum from a naturally infected cat. The third group ***A*** (Figure **6** **[A**], red) consisted also of a single grid node (4) with five sera from cats infected with non-canonical *T. gondii* strains, three sera from type I-infected cats and one serum from a type I infected cat.

Almost all sera from cats infected by clonal types I (n=11) and III (n=1) clustered in one unit (grid node ***5***) (Figure **6** **[C**]). Two of the remaining type *I sera* were grouped together with 15 sera obtained from naturally seropositive cats into grid node ***1*** (Figure **6** **[A**]**, [C**]). The remaining type I serum and four sera from naturally seropositive cats were placed in grid node ***2*** (Figure **6** **[A**]**, [C**]).

The serum of a type II infected cat clustered in grid node ***3*** together with 8.1% (n=7) of the sera obtained from naturally seropositive cats sampled in Germany (Figure **6** **[A**]**, [C**]). The second type II-specific serum was grouped with 36.04% (n=31) of the sera obtained from naturally-seropositive cats sampled in Germany and two sera from cats infected with non-canonical *T. gondii* in grid node ***6*** (Figure **6** **[A**]**, [C**]). The third type II serum was sorted together with 27.9% (n=24) of *T. gondii*-positive cat field sera into grid node ***7*** (Figure **6** **[A**]**, [C**]).

The remaining 5 of 7 sera collected from cats infected with non-canonical *T. gondii* clustered separately in grid node ***4*** together with one serum of a clonal type III-infected cat and three sera from cats infected with *T. gondii* clonal type I (Figure **6** **[A**]**, [C**]). 

In the grid node ***8*** only sera from naturally-seropositive cats (n=4) were present (Figure **6** **[A**]**, [C**]).

## Discussion

Several studies conducted with human and murine sera have shown that the antibody response against *T. gondii* is partially specific for the clonal type of the parasite [10,31]. Based on these findings non-invasive typing methods using synthetic peptides or recombinant polypeptides in serotyping ELISAs or microarrays were developed and used to investigate the presence of antibodies to specific clonal types of *T. gondii* in infected humans and animals [10,27,30,31,43,73]. To our knowledge, there is no published information about a clonal type-specific antibody response to *T. gondii* and serotyping in cats, although the cat plays a major role as an important definitive host in the epidemiology of the *T. gondii* infection. 

The availability of sera from individuals with a known clonal type of *T. gondii* infection is a prerequisite for developing serotyping tools. Since sera from humans with a known clonal type of *T. gondii* infection are very rare, sera from infected mice were used to evaluate peptides that were possibly suitable for serotyping with human sera [10]. Fortunately, we were in a position to use a few sera from experimentally infected cats to evaluate candidate peptides, i.e. to identify those peptides that showed optimal specificity for serotyping canonical type *T. gondii* infections. 

Some of the experimentally infected cats, whose sera were made available for the present study, had originally been used for oocysts production and were therefore orally inoculated with tissue cysts. It must be expected that this way of infection did not only induce the development of intestinal stages like schizonts and later-on gamonts, but also caused the establishment of extra-intestinal stages such as tachyzoites and bradyzoites [39]. As a consequence, cats shedding oocysts often develop a long-lasting humoral immune response against tachyzoites and bradyzoites. The resulting antibodies could be used for serotyping in order to determine the clonal type of *T. gondii* infection in individual cats. Since seroconversion to *T. gondii* is usually permanent, typing is not only possible in cats actually shedding oocysts (in Europe only a small proportion of less than 1% of all cats [21]) but also in the vast majority of animals that became infected with *T. gondii* in the past but failed to excrete oocysts or were not checked for oocyst shedding when they were bled. Serotyping may therefore allow a less biased view on the clonal types of *T. gondii* circulating in a cat population as compared to genotyping, which is necessarily restricted to a very small proportion of the cat population. 

In the present study, we analyzed a total of 101 *T. gondii* polymorphic peptides, i.e. peptides with clonal type specific aa sequences to identify molecules suitable for serotyping of *T. gondii* in cats. Sera from experimentally infected cats or cats, for which the type of the infecting *T. gondii* had been determined, as well as seronegative cats were used to select appropriate peptides, i.e. peptides with optimal specificity and sensitivity. From these 101 peptides, 54 had previously been characterized for *T. gondii* serotyping in humans [10]. Fifteen of the 54 peptides taken from the literature were shown to react in a clonal type-specific manner with cat sera. Another 47 peptides were selected for the present study using a bioinformatical approach that employed a propensity scale method [74]. Twenty-one of 47 (44.7%) peptides predicted by this approach revealed diagnostic capability (AUC value ≥ 0.7 in ROC analysis). However, only 9 of these 47 (19.1%) peptides were recognized in a clonal type-specific manner by cat sera. GRA5 derived peptides did not meet all the criteria of the propensity scale method. In spite of this, the success of a correct prediction of linear B-cell epitopes was similar in GRA5 peptides; i.e., 5 of 9 (55.6 %) GRA5 peptides revealed diagnostic capability and only 2 of 9 (22.2%) GRA5 peptides were recognized in a clonal type-specific manner. This illustrates an underperformance of the propensity scale approach to select appropriate peptides and corroborates the results of previous studies [75-77]. 

Interestingly, we found that peptides previously described as suitable for *T. gondii* serotyping in humans and mice [10,30,78] could also be used with feline sera. Obviously, such peptides contain clonal type-specific epitopes that are recognized by antibodies produced by a broad variety of vertebrate species including humans, mice and cats, and possibly also other intermediate hosts of *T. gondii*. In addition, these results show that resources available in both, human and veterinary medicine could be commonly used to improve sensitivity and specificity of serological tests as e.g. tests for serotyping. However, a large number of peptides showed a low clonal type-specificity in cats – although their aa sequences had indicated clonal type specificity – and were therefore not included into the final serotyping peptide panel. Similar results were obtained when peptides with polymorphic epitopes were validated with mouse sera [10]. The low type-specificity of particular peptides may be explained by a strong immune-reactivity in the non-polymorphic parts of these peptides. Further refinement of such peptides, e.g. by varying the length of their non-polymorphic portions may help to achieve type-specific reactivity [10].

Our experiments led to the detection of 24 peptides deemed suitable for typing canonical clonal types of *T. gondii* infection in cats using sera obtained from these animals. 

There was no reference serum from cats infected with canonical clonal types that failed to recognize peptides presenting epitopes with a homologous specificity for those *T. gondii* types the cats were infected with. We thus believe that this panel of polymorphic, clonal type-specific peptides can be used for epidemiological studies in cats in areas, where canonical types of *T. gondii* prevail, i.e. in Europe and North America. We applied the peptide panel to determine the distribution of the clonal types of *T. gondii* infection among naturally seropositive cats from Europe based on DNA from oocysts from naturally infected cats [20,21]. Genotyping and serotyping could therefore be compared for cats from the same region. 

Serotyping results in naturally seropositive cats revealed that clonal type II-specific peptides were recognized by the significantly highest MSIVs as compared to peptides with other sequence specificities. Moreover, type II-specific serum-peptide reactions were significantly overrepresented as compared to reactions with the remaining peptide groups. Explorative data analysis using XYF-SKN prediction revealed that 98.8% of sera (85/86) obtained from naturally seropositive cats clustered in one group and recognized similar peptide patterns as cats with a known *T. gondii* type II-infection, i.e. were assorted together with sera from in a single group (*N*) but into 6 different nodes suggesting slight differences in which peptides were recognized. Four peptides with type II specific aa sequences recognized by field sera were also recognized by cats with a confirmed or experimental *T. gondii* type II infection. However, in case of the peptide SAG2A-II-93 the reactions in cats with confirmed or experimental type II infection were only close to the cut-off while field sera reacted strongly with SAG2A-II-93. Differences in the expression of SAG2A between field and laboratory strains used for experimental infection in the present study, differences in the stage of infection between field and laboratory cats or host genetic factors could have contributed to this observation. Therefore, based on the serotyping results it is not unlikely that the majority of naturally seropositive cats from Germany were infected with type II *T. gondii*, which is in accord with genotyping results obtained with DNA from oocysts shed by cats [20,21] and with *T. gondii* serotyping results performed with human sera from Germany [30]. 

Although in the XYF-SKN analysis naturally seropositive cats were mainly assorted to nodes containing sera from type II-infected cats, also a few sera from cats infected with clonal types I (n=3) or cats infected with non-canonical *T. gondii* types (n=2) were assorted to these nodes. In addition, a few sera from cats experimentally infected with type I (3/16), type III (n=1/1) and atypical (5/7) strains clustered together in a single node in the XYF-SKN prediction, i.e. could not clearly separated from each other by serotyping. These results show the limitations of serotyping. As documented in Table **S4**, the specificity of some peptides for typing purposes is limited and it can be hypothesized that false positive reactions may occur in non-polymorphic, but also in the polymorphic (i.e. clonal type specific) regions of the peptides. These unspecific reactions in addition to a low number of reactive peptides may have attributed to the failure to differentiate between cats infected with canonical and atypical strains. In addition, it does not seem to be not possible to identify mixed infections unambiguously, as reactions with peptides with different type-specificities may have been caused by infections with more than one type (i.e. by mixed infections or superinfection) or by infection with an atypical strain.

Previous genotyping results suggested that mixed or superinfections with more than one canonical type of *T. gondii* can occur in cats under natural conditions [23,35], but there is no information on the expected frequency. It can be hypothesized that mixed or superinfection may be more likely in areas were more than one of the canonical types of *T. gondii* coexist. Since infection in cats seem to be clearly age-dependent [79], limiting serotyping to young cats (≤ 2 years of age) might be suitable to reduce the risk of misclassification due to mixed or superinfection in epidemiological studies.

Sousa et al. (2008) [29] reported on the serotyping in humans infected with non-canonical *T. gondii* types from Africa and South America by using peptides derived from clonal type II- and I/III-specific polymorphic GRA6 protein regions. Most of these human sera recognized mainly a clonal type I/III GRA6 peptide [29]. In the present study, we serotyped cats infected with non-canonical *T. gondii* from Brazil (TgCatBr1, 2, 5) and North America (TgBbUS1 and TgGoatUS6). Interestingly, these sera also recognized clonal type I/III-specific peptides with the significantly highest mean MSIVs and the number of positive type I/III peptides reactions were significantly overrepresented in these sera. Strong reactions with individual peptides specific for clonal types I, II or III were also observed. The strong IgG response against type II-, or type I/III-specific peptides in cats infected with non-canonical *T. gondii* may indicate that especially proteins with epitopes common in type II, or I/III are expressed by these non-canonical strains. Since sequences of proteins from these atypical *T. gondii* isolates are not available, it was not possible to test this hypothesis. Although cats infected with atypical *T. gondii* reacted strongly with peptides specific at the same time for type I and III (i.e. type I/III peptides) most of these cats showed peptide patterns different from cats infected with type I and type II (Figure **6**). This finding provides confidence that our serotyping assay would have been able to detect during field study a population of cats with non-canonical *T. gondii* infection. However, both, mixed infections and infections with non-canonical or atypical strains (as shown in the present study) may cause an antibody response reactive with single peptides containing epitopes specific for canonical types of *T. gondii*. Therefore, serotyping results have to be analyzed with care to avoid misinterpretation. We therefore propose serotyping as an epidemiological tool for typing in areas where canonical *T. gondii*-infections prevail, but not as a tool for typing individual cats. 

In conclusion, in the present study we showed that cats mount an antibody response that is specific for the *T. gondii* clonal type and identified 24 peptides suitable for *T. gondii* serotyping in cats. Our results suggest that most *T. gondii* seropositive cats in Germany are infected with type II *T. gondii*. This finding is in accord with previous findings reporting a predominance of type II *T. gondii* in oocysts shed by cats in Germany [20] and with *T. gondii* serotyping in humans from Germany, which also suggested that type II infections prevailed [30]. Further studies with larger numbers of well-defined sera may help to improve the evaluation of polymorphic peptides and to identify more peptides suitable for *T. gondii* serotyping in cats. 

## Supporting Information

Table S1 **Polymorphic peptides based on protein sequences available for *Toxoplasma gondii* antigens.**
(XLS)

Table S2 **Cat reference sera used to evaluate and select peptides for the serotyping of *Toxoplasma gondii*.**
(XLS)

Table S3 **Corrected mean sample index values (signal intensity) listed for all peptides and sera.**
(XLS)

Table S4 **Diagnostic parameters of peptides used in this study.**
(XLS)
